# Ubiquitin Specific Peptidase 15 (USP15) suppresses glioblastoma cell growth via stabilization of HECTD1 E3 ligase attenuating WNT pathway activity

**DOI:** 10.18632/oncotarget.22798

**Published:** 2017-11-30

**Authors:** Maria Oikonomaki, Pierre Bady, Monika E. Hegi

**Affiliations:** ^1^ Department of Clinical Neurosciences, University Hospital Lausanne, Lausanne, Switzerland; ^2^ Department of Research and Education, University Hospital Lausanne, Lausanne, Switzerland; ^3^ Bioinformatics Core Facility, SIB Swiss Institute of Bioinformatics, Lausanne, Switzerland

**Keywords:** glioblastoma, USP15, HECTD1, tumor suppressor, WNT pathway

## Abstract

Expression based prediction of new genomic alterations in glioblastoma identified the de-ubiquitinase Ubiquitin Specific Peptidase 15 (*USP15*) as potential tumor suppressor gene associated with genomic deletions (11%). Ectopic expression of USP15 in glioblastoma cell-lines reduced colony formation and growth in soft agar, while overexpression of its functional mutant had the opposite effect. Evaluation of the protein binding network of USP15 by Mass Spectrometry in glioblastoma cells uncovered eight novel interacting proteins, including HECT Domain Containing E3 Ubiquitin Protein Ligase 1 (HECTD1), whose mouse homologue has been associated with an inhibitory effect on the WNT-pathway. USP15 de-ubiquitinated and thereby stabilized HECTD1 in glioblastoma cells, while depletion of USP15 led to decreased HECTD1 protein levels. Expression of USP15 in glioblastoma cells attenuated WNT-pathway activity, while expression of the functional mutant enhanced the activity. Modulation of HECTD1 expression pheno-copied the effects observed for USP15. In accordance, human glioblastoma display a weak but significant negative correlation between *USP15* and *AXIN2* expression. Taken together, the data provide evidence that USP15 attenuates the canonical WNT pathway mediated by stabilization of HECTD1, supporting a tumor suppressing role of USP15 in a subset of glioblastoma.

## INTRODUCTION

Glioblastoma (GBM) is the most malignant primary brain tumor in adults with a dismal prognosis of only 15 to 18 months [1]. The tumor is refractory to most therapies, likely due to a plethora of genetic and epigenetic alterations affecting multiple cancer relevant pathways conferring enhanced adaptive plasticity to tumor cells [2, 3]. Interestingly, so far no strong addiction to single oncogene activation has been identified that was successfully druggable in patients. In a screen of a cohort of GBM for aberrantly expressed genes to infer underlying molecular alterations, we identified *Ubiquitin Specific Peptidase 15 (USP15)* (12q14.1) to be associated with genomic deletions, suggestive of a potential tumor suppressing function in GBM [4]. The region 12q14-15, comprising the *USP15* locus, has been identified as a breakpoint rich region (BER) affecting a subset of GBM. BER results in complex patterns of gene alterations, including deletions, amplifications, rearrangements, and fusions that have been associated with worse outcome [5]. This chromosomal region is flanked by the proto-oncogenes *CDK4* and *MDM2* that are amplified individually or together in a subset of GBM, and co-amplification overlaps significantly with BER.

USP15 belongs to the ubiquitin-specific protease (USP) family, which is the largest sub-group of de-ubiquitinase enzymes (DUBs), and exerts cysteine endopeptidase activity [6]. Deregulation in the ubiquitination machinery has been associated with several types of cancer and may serve as target for cancer therapy [7]. The function of USP15 has been mainly associated with the COP9 signalosome (CSN). CSN is a conserved multiprotein complex typically consisting of eight subunits designated as CSN1-CSN8 [8]. USP15 is emerging as a multifunctional DUB regulating also multiple proteins involved in cancer relevant pathways thereby mediating both, tumor suppressing and oncogenic functions in a context dependent manner. Oncogenic properties have been reported from ovarian cancer, where USP15 stabilizes HPV16E6 [9], or from divers cancer cell lines where USP15 stabilizes the oncogene REST [10] or MDM2 [11], respectively. Further, an oncogenic role has been reported through interaction and stabilization of the TGF-β receptor I, potentially activating TGF-β signaling in a small subgroup (2%) of GBM [12]. On the other hand reports on tumor suppressing functions comprise the stabilization of p53 with subsequent induction of p21 [13], inhibition of EB1 by interacting and stabilizing the E3 ligase that ubiquitinates EB1 [14], stabilization of APC, thereby attenuating the WNT signalling pathway [15], or stabilization of procaspase, promoting caspase 3 mediated apoptosis. Furthermore, the *USP15* gene has been shown to be deleted in 25.37% of pancreatic cancer [16].

Here we investigated the function of USP15 in GBM by identifying its interaction partners, and determining their function in GBM cells.

## RESULTS

Evaluation of gene copy number aberrations of *USP15* in GBM revealed a deletion frequency of 10.9% (95% exact confidence interval [CI] 4.5-21.2) for our GBM dataset (NCH-EORTC, n= 64, human methylation 450K BeadChip) which was corroborated in the GBM dataset of The Cancer Genome Atlas (TCGA) with a deletion frequency of 10.8% (95% [CI] 8.0-14.2) and an amplification frequency of 1.2% (95% CI, 0.4-2.8) (n=415, ACGH-244k) [2].

### USP15 overexpression inhibits cell proliferation and growth in soft agar

**Figure 1:**
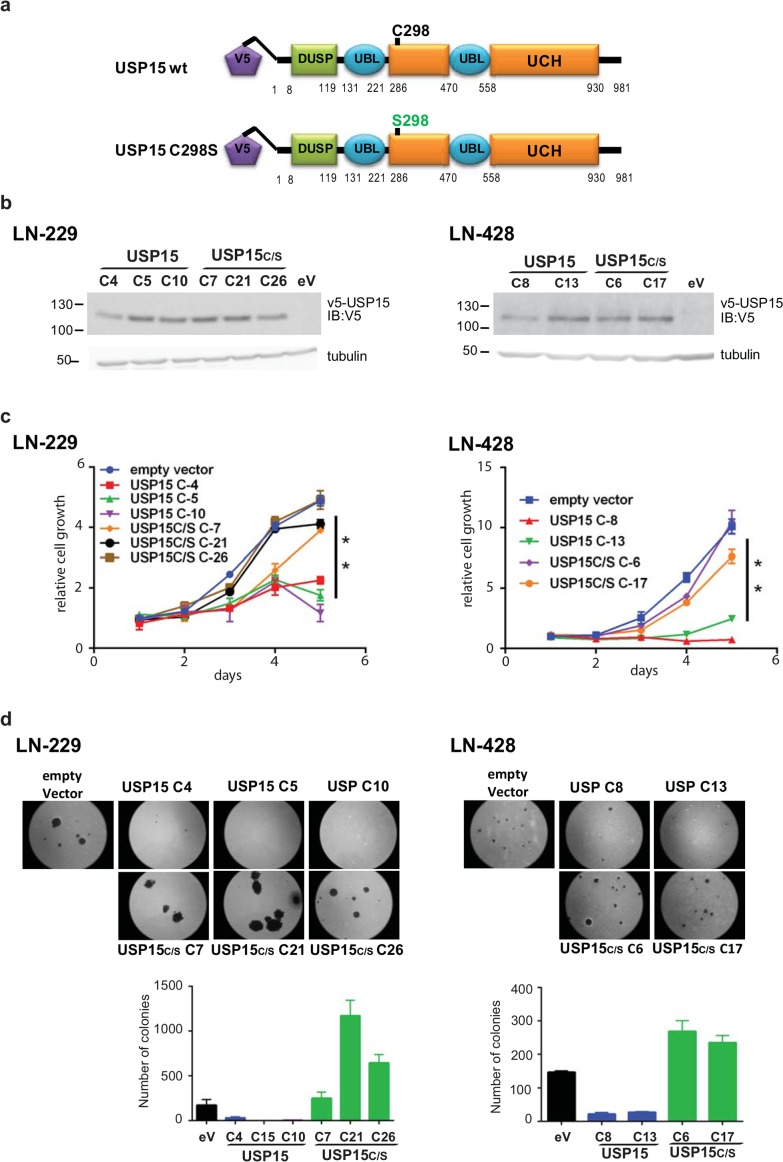
Effect of USP15 on cell growth in LN-229 **(a)** Structure of the wildtype USP15 construct with the V5 protein tag, and the respective catalytic mutant USP15^C298S^. **(b)** Protein expression by Western blot using an anti-V5 AB, loading control tubulin for the selected clones from LN-229 and LN-428 with stable expression of USP15, or USP15^C298S^ (USPC/S), or empty vector controls. **(c)** Growth of USP15, or USP15^C298S^ (USPC/S) overexpressing clones, and respective empty vector control (pIRES2/EGFP) followed over 5 days in culture. The error bars indicate standard deviations of triplicate samples. **(d)** Adhesion-independent growth in soft agar, representative images, and quantification of total colony numbers. Empty vector control, eV, black; USP15, blue; USP15C/S, green. Histogram shows data as mean +/-SD.

In order to evaluate the effect of USP15 in human GBM, we stably transfected the GBM cell lines LN-229 and LN-428 to ectopically express USP15 or its catalytic mutant USP15^C298S^, using a pIRES2-EGFP backbone with a V5 tag. Cysteine 298 is in the catalytic site (catalytic triad) of the protein, and mutation into serine abrogates the ability to de-ubiquinate proteins [17]. The selected clones were subjected to a proliferation assay over 5 days under standard conditions. Cell growth of USP15 overexpressing clones and clones expressing the mutant, USP15^C298S^, or corresponding EGFP-vector controls was significantly different (t-test, p-value < 0.01) in both GBM cell lines. The USP15 overexpressing clones grew less than the other clones (Figure 1). Furthermore, adhesion-independent cell growth as tested in the soft agar assay showed even more striking results. After 3 weeks of culture in soft agar the USP15 overexpressing cells displayed a reduced ability to form colonies as compared to the USP15^C298S^ clones and the EGFP-vector controls in both cell lines (Figure 1). The LN-229 derived USP15 expressing clones even completely lost the ability to grow in soft agar. Interestingly, the clones expressing the mutant USP15^C298S^ formed more and bigger colonies in soft agar than the EGFP-vector controls (Figure 1). This observation may suggest that the USP15 mutant USP15^C298S^ out-competes the endogenous USP15 leading to enhanced cell proliferation in LN-229 and LN-428 *in vitro*.

### USP15 protein binding network in LN-229 GBM cells

**Figure 2:**
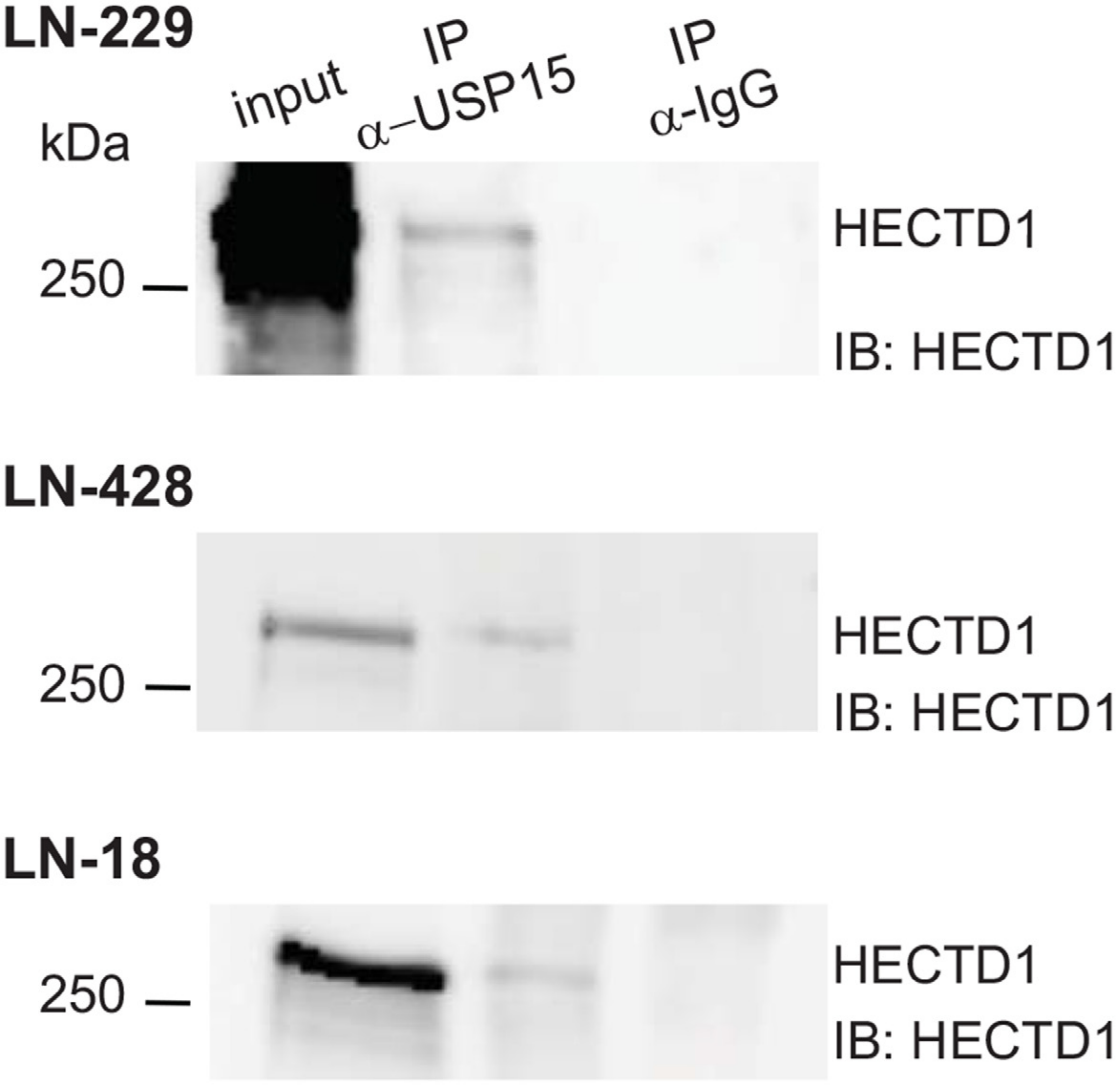
USP15 interacts with HECTD1 in GBM cell lines Immunoprecipitation using an antibody against the endogenous USP15 in LN-229, LN-428, and LN-18 protein extracts, analyzed by Western blot using an antibody against HECTD1. The control immunoprecipitation was performed with anti-rabbit IgG.

In order to identify GBM-relevant molecular pathways in which USP15 is implicated, we aimed at identifying its protein binding partners in the GBM cell line LN-229. Immunoprecipitation (IP) of endogenous USP15 in LN-229 was followed by mass spectrometry (MS). In the control experiment an antibody against rabbit IgG was used. Fractions of the immunoprecipitated proteins were analyzed by Western blot and by SDS-PAGE (silver staining) to confirm that the endogenous USP15 was expressed at a sufficient level for detection by MS. MS identified 502 proteins. From the top 11 proteins with 7 or more spectra and none in the IgG control, 8 were confirmed in an independent IP/MS experiment (Table 1). From these top proteins three were known interactors of USP15: Squamous Cell Carcinoma Antigen Recognized By T Cells 3 (SART3) [18, 19, 20], USP11 [21, 22] and USP4 [22], suggesting that IP-MS analysis worked efficiently. We selected four of the novel potential USP15 binding partners HECT Domain Containing E3 Ubiquitin Protein Ligase 1 (HECTD1), Oxysterol Binding Protein-Like 3 (OSBPL3), and Kinesin Family Member 15 (KIF15), Regulator Of Microtubule Dynamics 3 (RMDN3) that seem to be implicated in cancer relevant molecular mechanisms [23–30]. The interactions were validated by Western using the respective specific antibodies after loading proteins immunoprecipitated from LN-229 protein extracts with the AB against the endogenous USP15, or the control AB against rabbit IgG, respectively (Figure 2, Supplementary Figure 1).

**Table 1:** USP15 Interactors in glioblastoma cell lineLN-229

Identifier	UniProt description	Number of spectra			
		1^st^ IP		2^nd^ IP	
		α-USP15	IgG control	α-USP15	IgG control
**USP15**	Ubiquitin carboxyl-terminal hydrolase 15	232	2	392	0
**SART3**	Squamous cell carcinoma antigen recognized by T-cells 3	56	0	97	0
**HECTD1**	E3 ubiquitin-protein ligase HECTD1	25	0	68	0
**OSBPL3**	Oxysterol-binding protein-related protein 3	18	0	48	2
**KIF15**	Kinesin-like protein KIF15	17	0	41	0
**DRP1**	Dynamin-1-like protein	10	0	22	0
**UBP11**	Ubiquitin carboxyl-terminal hydrolase 11	14	0	24	0
**UBP4**	Ubiquitin carboxyl-terminal hydrolase 4	14	0	23	0
**RMD3**	Regulator of microtubule dynamics protein 3	11	0	11	0
**GLNA**	Glutamine synthetase	9	0	19	0
**CO4A**	Complement C4-A	7	0	10	0

### Interaction of USP15 with HECTD1

We then selected HECTD1 for further analyses. HECTD1 had the most spectra of the new binding partners in both experiments (Table 1). Furthermore, a recent report indicated that the murine homologue, Hectd1 is a negative regulator of the Wnt pathway [31]. Hectd1 was shown to tag Adenomatous polyposis coli (APC) with a Lys-63 linked ubiquitin chain in HEK293 cells, thereby promoting APC-Axin interaction, leading to negative regulation of Wnt signaling. This Lys-63 ubiquitinilation of APC by Hectd1 is reversible by the de-ubiquitinase Trabid, resulting in Wnt pathway activation. Additionally, it was shown that the Trabid-Hectd1-APC complex interacts with proteins composing the striatin-interacting phosphatase and kinase (STRIPAK) complex that regulates cortical actin cytoskeleton dynamics [31]. Interestingly, our IP-MS analyses comprised proteins from the STRIPAK complex (Supplementary Table 1).

**Figure 3:**
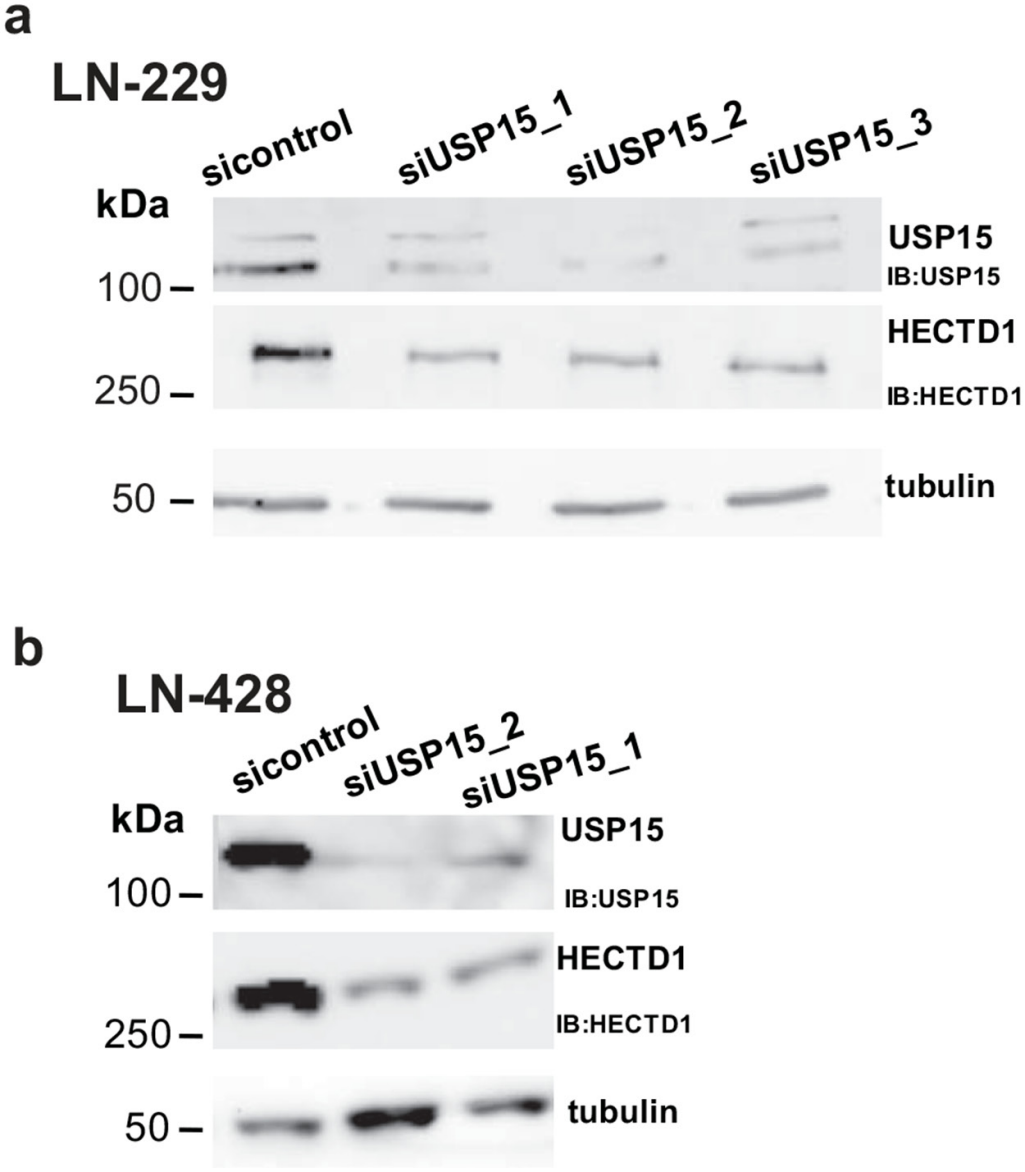
USP15 knockdown leads to decreased HECTD1 protein levels **(a)** USP15 knockdown with three siRNAs in LN-229 and **(b)** two siRNAs in LN-428.

First we confirmed the interaction between USP15 and HECTD1 in two additional GBM cell lines, LN-428 and LN-18 after immunoprecipitation of the endogenous USP15 by Western blot (Figure 2). Then we assessed the nature of the interaction between USP15 and HECTD1. Depletion of USP15 by siRNAs in LN-229 and LN-428 resulted in a reduction of HECTD1 protein as determined by Western blot (Figure 3), suggesting a stabilizing effect of USP15 on HECTD1.

### USP15 stabilizes E3 ligase HECTD1 via de-ubiquitination

**Figure 4:**
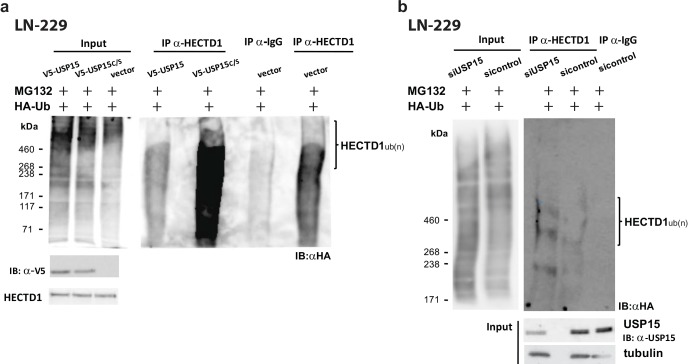
USP15 de-ubiquitinates and stabilizes HECTD1 in LN-229 The de-ubiquitination assay was performed in cells transfected with an expression plasmid for HA-Ubiquitin that 40 hours after transfection were treated for 8 hours with the proteasome inhibitor MG132 (5μM). The cells were lyzed and used for IP using an antibody against the endogenous HECTD1, or an AB against rabbit IgG in the negative control. The immunoprecipitated proteins were then analyzed by Western blot using an anti HA antibody to detect ubiquitination. **(a)** LN-229 cells stably expressing USP15, the mutant USP15C/S, or the empty vector control, respectively. Lanes 1 to 3 correspond to the input controls. The USP15 wt overexpressing LN-229 cells show lower ubiquitination levels of HECTD1 than the vector control. The mutant USP15-C/S overexpressing cells displayed the highest ubiquitination of HECTD1. **(b)** De-ubiquitination assay in LN-229 depleted for endogenous USP15 by siRNA against USP15 or the respective si-control. LN-229 cells depleted for endogenous USP15 display higher ubiquitination of HECTD1 than the sicontrol cells.

To better understand the USP15-HECTD1 interaction we performed a de-ubiquitination assay. To this end, LN-229 cells ectopically expressing either USP15, the functional mutant USP15^C298S^, or the EGFP-vector control, were transfected with a HA-Ubiquitin construct. Forty hours later cells were treated with 5μM of the proteasome inhibitor MG132 for 8 hours. Cells were lyzed and used for immunoprecipitation using an antibody against the endogenous HECTD1. The immunoprecipitated proteins were analyzed by Western blot using an anti-HA antibody. Western blot analysis revealed that overexpression of USP15 in LN-229 cells caused a reduction in HECTD1 ubiquitination, whereas USP15^C298S^ increased HECTD1 ubiquitination (Figure 4). Again, suggesting that the mutant form out-competed the endogenous USP15. In accordance, the de-ubiquitination assay in LN-229 cells that were transfected with siUSP15, showed that knockdown of endogenous USP15 increased the incorporation of ubiquitin into HECTD1, as compared to the siRNA control cells (Figure 4). Taken together, these data suggest that USP15 is able to cleave the ub-conjugates from HECTD1, thereby rescuing it from proteasome degradation, leading to stabilization of the protein. However, direct interaction remains to be shown.

### HECTD1 overexpression in LN-229 cell lines inhibits growth in soft agar

**Figure 5:**
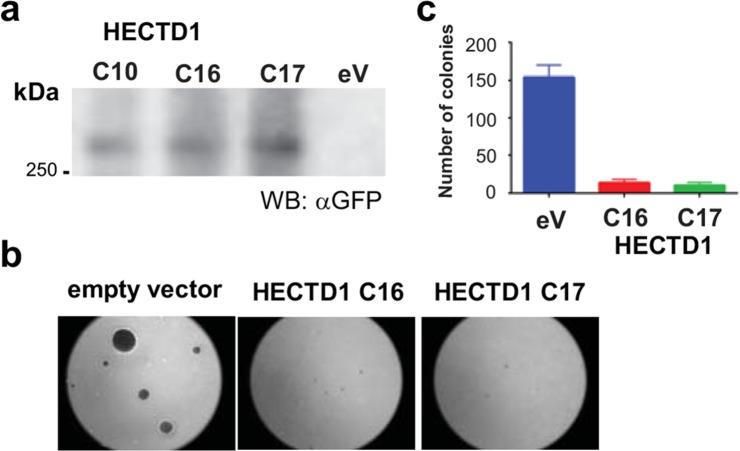
Effect of HECTD1 on cell growth LN-229 clones stably overexpressing HECTD1 as determined by Western blot (respective RNA expression levels of *HECTD1* [ectopic and endogenous] are shown in Figure 6c) **(a)** were tested for adhesion-independent growth in soft agar **(b)**. Quantification of colonies revealed strong reduction of colony formation in HECTD1 overexpressing cells **(c)** (data as mean +/-SD).

Next, we aimed at elucidating the role of HECTD1 in GBM. We established LN-229 cells that stably express GFP-HECTD1 as confirmed by Western blot (Figure 5). We tested the effect of HECTD1 on growth in soft agar. HECTD1 overexpressing clones displayed a strikingly reduced ability to form colonies as compared to the EGFP-vector control cells after 3 weeks (Figure 5). The empty vector carrying cells displayed 90-94% more colonies as compared to the HECTD1 overexpressing clones (Figure 5). Consistent with this finding, depletion of endogenous HECTD1 by siRNAs resulted in an acceleration of the cell cycle as determined by fluorescence-activated cell sorting (FACS) (Supplementary Figure 2).

### HECTD1 and USP15 have a negative effect on WNT pathway activity

**Figure 6:**
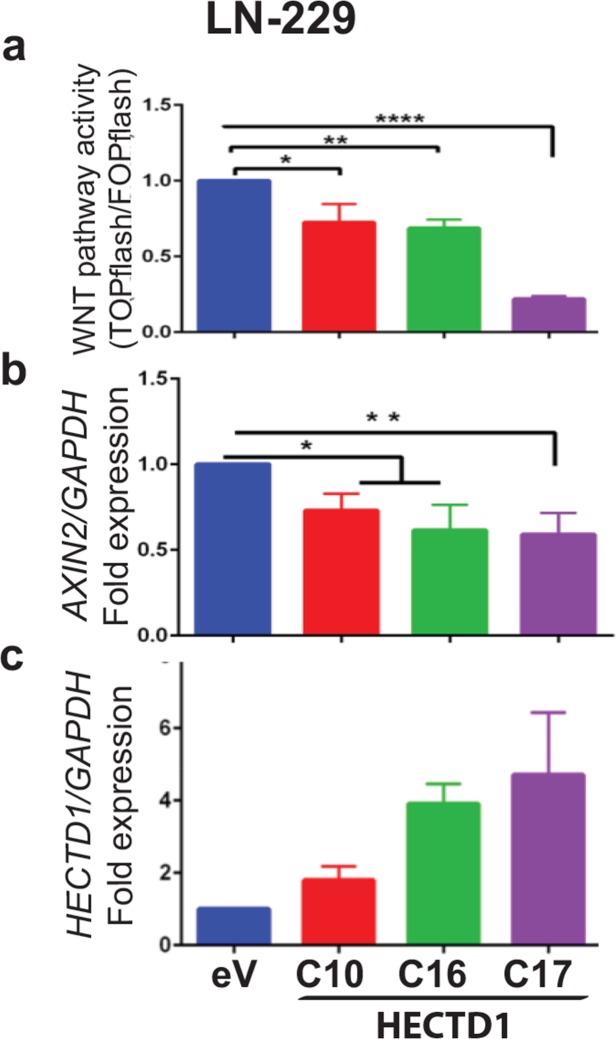
HECTD1 downregulates WNT pathway activity in LN-229 **(a)** WNT pathway activity was measured with the TCF luciferase reporter system (TOPflash/FOPflash) in LN-229 clones stably expressing HECTD1 and the empty vector control cells. The error bars indicate standard deviation of three independent experiments. *AXIN2* **(b)** and *HECTD1* **(c)** expression were measured by qRT-PCR relative to GAPDH. The histograms shown are representative of three independent experiments (data as mean +/-SD). The experiments are normalized to the empty vector control (eV).

**Figure 7:**
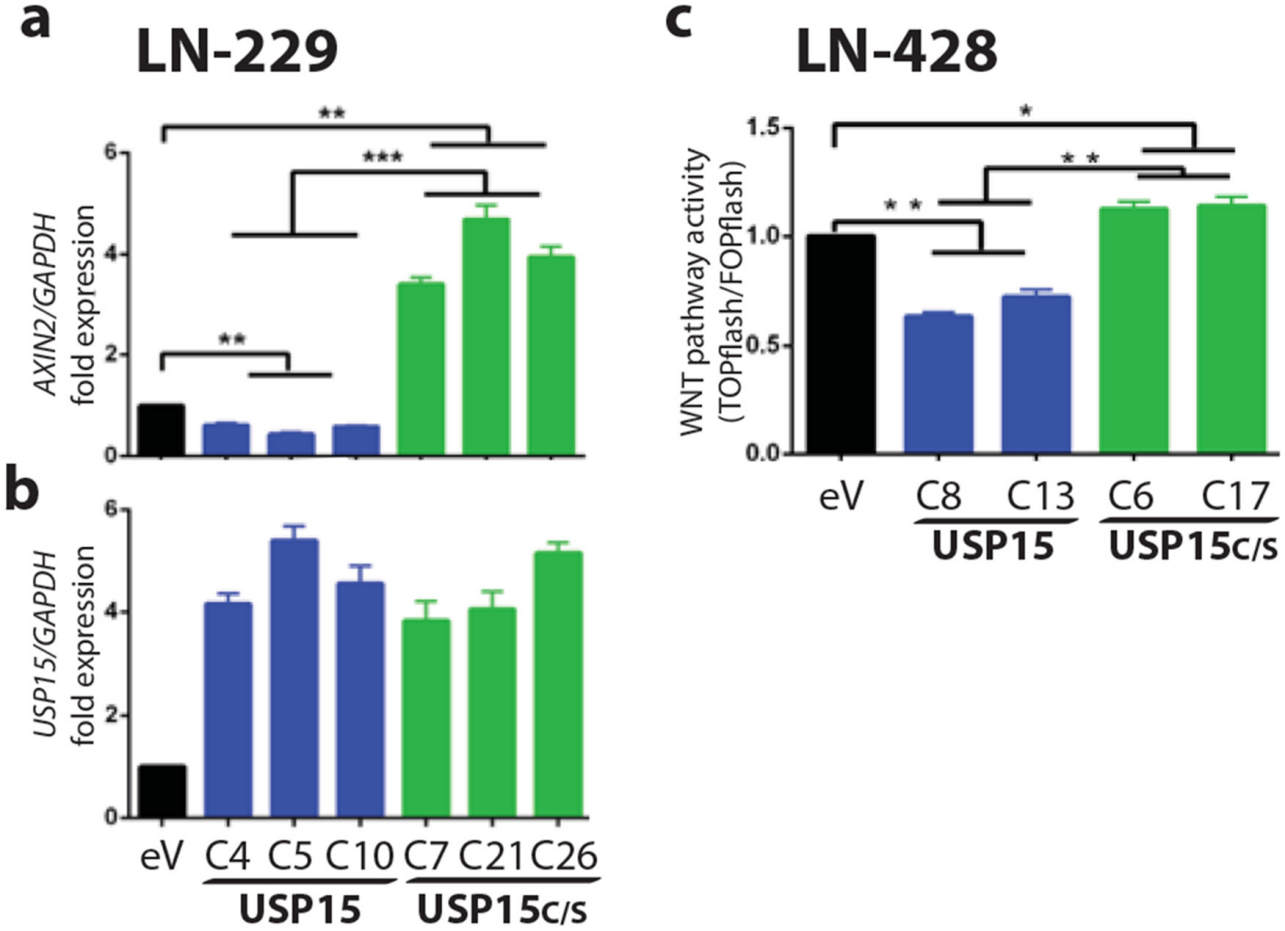
USP15 exerts an inhibitory effect on WNT pathway activity in GBM cell lines **(a)** *Axin2* is canonical WNT-pathway target gene and its expression was measured as read-out for WNT-pathway activity. qRT-PCR of *Axin2* relative to *GAPDH* was peformed in LN-229 clones stably expressing *USP15* (blue), the functional mutant *USP15C/S* (green), or the empty vector control cells (black). **(b)** Expression of *USP15* relative to *GAPDH*. **(c)** Canonical WNT pathway activity was measured with the TCF/LEF luciferase reporter system (TOPflash/FOPflash) in LN-428 clones stably expressing *USP15* (blue), *USP15C/S* (green), or the empty vector control. Histograms are representative of two independent experiments (data shown as mean +/-SD). In both cell lines overexpression of USP15 attenuated WNT-pathway activity, while the functional mutant USP15C/S displayed an activating effect. The experiments are normalized to the empty vector control (eV).

Since the murine homologue of HECTD1 has been shown to interact with APC in HEK293 cells promoting the APC-Axin interaction and leading to negative regulation of Wnt signalling [31] we determined the effect of HECTD1 on the WNT pathway using the HECTD1 expressing LN-229 cell clones. The activation of the β-catenin dependent pathway was measured by the TCF/LEF reporter system (TOPflash/FOPflash luciferase assay). HECTD1 overexpressing LN-229 cells displayed reduced WNT pathway activity as compared to the respective EGFP-vector control cells (Figure 6). In accordance, differential qRT-PCR analysis revealed that HECTD1 overexpressing clones had significantly lower *AXIN2* gene expression. *AXIN2* is a direct canonical WNT-pathway target gene, mediated through TCF/LEF factors, whose expression reflects canonical WNT-pathway activity [32, 33]. The decreased *AXIN2* expression thus supports the notion that HECTD1 attenuated the WNT pathway (Figure 6). We then went back to test the effect of USP15 on WNT pathway activity by measuring *AXIN2* expression as read-out in LN-229. The USP15 overepxressing clones displayed significantly reduced levels of *AXIN2* expression as compared to the empty vector control, while the clones expressing the mutant USP15^C298S^ exerted incereased expression (Figure 7a). We then confirmed the negative regulatory effect of USP15 on the WNT pathway in the LN-428 GBM cell line using the TCF/LEF WNT-pathway luciferase reporter. Compared to the EGFP-vector control, the USP15 overexpressing clones displayed a significantly attenuated WNT pathway activity, while the mutant USP15^C298S^ overexpressing clones displayed a modest increase (Figure 7c).

Taken together, the data suggest that USP15 as well as HECTD1 are involved in the negative regulation of the WNT pathway in GBM.

### USP15 and the Wnt pathway in human GBM

**Figure 8:**
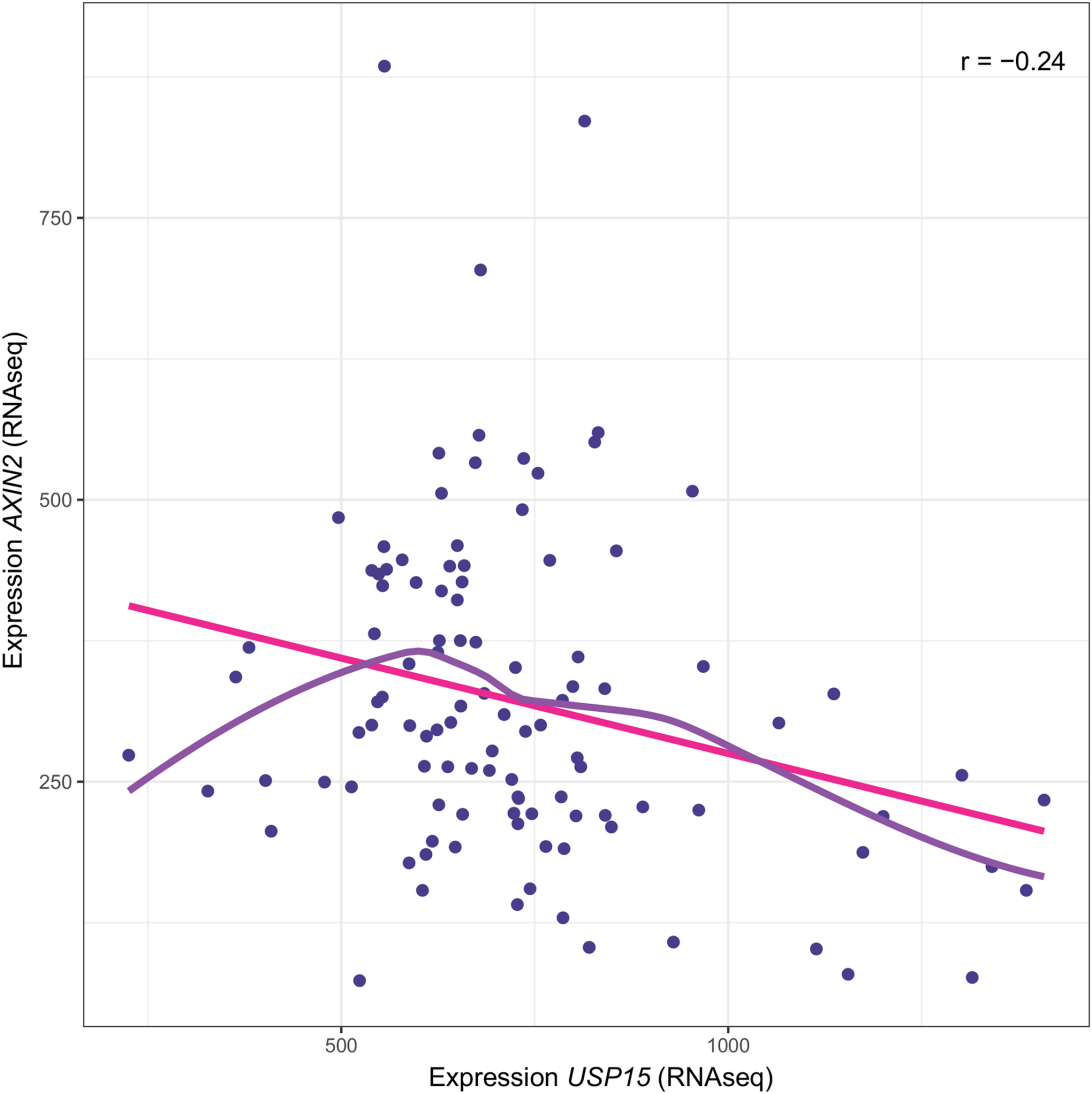
Association of USP15 expression and the WNT target gene AXIN2 in human GBM Negative correlation between expression of *USP15* and *AXIN2* in the TCGA dataset (r=-0.24 spearman correlation; p=0.01, Monte-Carlo test, 99 permutations; n=107) for which RNAseq data is available. The solid pink line corresponds to the linear model (y= a +xb) and the purple line to the LOWESS smoother (locally-weighted polynomial regression [48]).

In order to evaluate whether the here delineated regulation of the WNT pathway through USP15 is of relevance in human GBM, we interrogated the relationship between expression of *USP15* and the prototypic WNT target gene *AXIN2* in human GBM samples using the TCGA dataset (n=107; normalized level 3 RNAseq data). A significant negative correlation was observed (Spearman correlation coefficient r=-0.24; Monte-Carlo test, p=0.01 for 99 permutations) (Figure 8). Hence the data from GBM is compatible with the hypothesis that USP15 may exert a negative regulatory effect on the WNT pathway in GBM.

## DISCUSSION

Our previous analyses of GBM datasets have pointed to the de-ubiquitinase *USP15* as a candidate tumor suppressor gene for GBM based on gene expression and gene CNV patterns [4]. Functional evaluation of USP15 by ectopic modulation of expression in GBM cell lines established an inhibitory effect on cell proliferation that was more remarkable under non-adherent conditions, while overexpression of the functional mutant enhanced cell growth under these conditions. USP15 is a multifunctional DUB whose context dependent function is mediated by the presence of cell specific substrates involved in multiple biologic mechanisms including cancer relevant pathways. Interestingly, these involve reportedly both tumor suppressing, but also oncogenic functions [9, 10, 11, 12, 13, 15, 34–37]. Exploration of the glioblastoma specific interactome of USP15 indentified HECTD1 as novel top binding partner. HECTD1 attracted our attention for its reported involvement in the negative regulation of the WNT pathway through ubiquitination of APC, promoting APC-Axin interaction [31]. We have demonstrated that downregulation of USP15 in GBM cells reduced HECTD1 levels, and that HECTD1 was substrate for de-ubiquinitation by USP15, but not by its functional mutant, suggesting that USP15 stabilizes HECTD1. We showed that HECTD1 negatively regulates canonical WNT activity in GBM cells similar to USP15, as determined by reporter assay or measuring expression of the prototypic WNT target gene *AXIN2*. Modulation of HECTD1 expression pheno-copied the effects of USP15 in GBM cells. From these data we concluded that USP15 may exert its negative regulatory effect on WNT pathway activity through stabilizing HECTD1.

The significant negative association of *USP15* expression with the canonical WNT pathway target gene *AXIN2* in a human GBM dataset is consistant with a negative regulatory role of USP15 as proposed by our *in vitro* study. Hence, USP15 may indeed have a tumor suppressive role that is targeted in a subset of GBM by deletion (11%), resulting in enhanced WNT pathway activity. This adds a novel mechanism of WNT pathway deregulation observed in GBM that include genetic and epigenetic inactivation of negative regulators, such as Dickkopf, the family of Secreted Frizzled-Related Proteins (SFRPs), and the WNT inhibitory factor 1 (WIF1) that affect the canonical, and in the case of WIF1 also the non-canonical WNT pathway [4, 33, 38, 39]. This new mechanism of WNT pathway deregulation emphasizes the importance of active WNT signalling for GBM biology. Ubiquitination and de-ubiquitination are interesting targets for drug development, especially in cancer [40]. Further study of the regulation of this USP15 interaction or the potential binding network of HECTD1 may allow development of new treatment strategies.

## MATERIALS AND METHODS

### Datasets, gene copy number analyses, RNAseq analyses

Gene copy number analyses was performed using human methylation 450K data from the NCH-EORTC glioblastoma dataset (n=64; GEO accession number GSE60274) [41] and ACGH-244k from TCGA (n=415) [42]. A mixture model using the R package CGHcall was used for classification as detailed previously [43]. The Spearman ‘rank’ correlation between two genes was calculated using level 3 RNAseq data from the TCGA GBM dataset (n=107) [42] and tested for significance using the Monte-Carlo test (99 permutations) [44]. The dbGaP accession number to the specific version of the TCGA data set is phs000178.v8.p7.

### Glioblastoma cell lines

The GBM cell lines LN-229, LN-428, and LN-18 have been established in our laboratory and were authenticated by DNA fingerprinting [45]. Cell lines were cultured in Dulbecco's modified Eagle's medium (Invitrogen, USA), supplemented with 5% fetal calf serum (Hyclone, USA) and 100 units/mL penicillin, 100 units/mL streptomycin (Invitrogen, USA).

### Plasmid construction and small interfering RNAs

The full-length pIRES2-EGFP-V5-USP15 was subcloned as follows: DNA fragments encompassing residues 1–1627 and 1628-3000 of the human USP15 isoform 1 were amplified by platinum TaqDNA Polymerase High Fidelity (Invitrogen, USA) using a cDNA template derived from the LN-229 cell line using primers described in Supplementary Table 2. The first product was digested with Sal1 (Roche, Germany) and PST1 (NEB, USA), while the second was digested with Pst1 (NEB) and Xba1 (Invitrogen). The products were subjected to a triple T4 DNA ligation reaction (Promega, USA) overnight at 4°C together with the Stag-V5 pcDNA3 vector that had been digested with Xho1 and Xba1. The Stag-V5 pcDNA3 vector carries the streptavidin (Stag) and V5 tag and was kindly provided by Prof. Widmann (University of Lausanne). Subsequently, the insert was subcloned into pBluescript SKII(+) (Stratagene) between the HindIII/Xba1 restriction sites, in order to add the polylinker. The full length V5-USP15 was cloned into the pIRESII-EGFP vector (Clontech, USA) between the HindIII/SacII restriction sites.

The catalytic mutant of USP15 (USP15C/S) was created by PCR mutagenesis using as a template the intermediate V5-USP15/pBluescript SKII construct and the primers described in Supplementary Table 2. After PCR, the product was subjected to Dpn1 digestion for the destruction of the initial plasmid template. The isolated mutated product was cloned into the pIRESII-EGFP vector between the HindIII/SacII restriction sites.

The pEGFP-HECTD1 construct was kindly provided by Cai Huang [46] and the HA-Ubiquitin by Dr. Phil Shaw (University Hospital Lausanne). The Wnt/β-catenin activity luciferase reporters TOP_FLASH and FOP_FLASH comprise T-cell factor (TCF)/β-catenin responsive elements that express synthetic firefly luciferase from a PGL4.10 backbone with a minimal TATA box with 8 concatenated TCF binding sites, and 8 mutated binding sites, respectively (kindly provided by Prof. Tatiana Petrova, University of Lausanne) [47]. The pRL CMV Renilla luciferase (Promega AG) plasmid was used to normalize for transfection efficiency. All constructs were validated by sequencing (Microsynth, Switzerland). For transformation, bacteria (Ecoli strain DH5a9) were incubated with the plasmids for 30 minutes on ice, and subjected to heat shock for 45 s at 42°C. Bacteria were put on ice for 2minutes, and then left to recover in Luria Bertani (LB, Sigma, USA) liquid media without antibiotic for 45minutes at 37°C in a shaking incubator. Later bacteria were plated on petri dish with solid LB-agar (Fluka, USA) and the antibiotic for selection (ampicillin, kanamycin, Sigma).

Plasmid purification from liquid bacteria culture was performed with the Qiafilter Plasmid Midi kit (Qiagen, Netherlands), or the Qiaprep Spin Miniprep kit (Qiagen).

### Cell transfection and establishment of stable cell lines

Cell lines were transfected with the system NEON electroporator (invitrogen) at 1400 Volts, 20 Width, and 1 pulse using 100 μl tips. A ratio of 15μg of DNA/1*10^6 cells was used, while the final concentration of transfected siRNA was 50 nM (sequences Supplementary Table 2). For stable transfections, cells were selected with G418 (400-800 μg/mL). Resistant clones were selected, and isolated by cell sorting. Sorted cells were subjected to single clone culture and maintained under G418.

### Crystal violet assay

Growth curves were performed in 12-well plates. In each well, 2-3 × 10^4^ cells were seeded. At every time point, culture medium was removed, cells were washed with 1mL of 1X phosphate buffered saline (PBS), and 500 μL of crystal violet solution was added per well. After 10 min, crystal violet solution was removed and plates were washed with 1× PBS. Plates were left to dry, and 500 μL of 1% sodium dodecyl sulfate (SDS) in distilled water was added per well. Absorbance was then measured at 595 nm using a plate reader.

### Colony formation assay in soft agar

Soft agar assay was performed in 6-well plates in 2mL of 1% agar in complete medium as the bottom layer, and 1 mL of 0.4% agar in complete medium as the top layer. 2 × 10^3^ cells were seeded in triplicate. Cultures were maintained under standard conditions, and after 3 weeks colonies were stained with 2-(4-iodophenyl)-3-(4-nitrophenyl)-5-phenyl-2H-tetrazolium chloride (INT) (Sigma). The colonies were detected with an inverted phase-contrast microscope, where a group of 50 cells was scored as colony. Colonies were photographed and quantified by ImageJ program.

### Immunoprecipitation and mass spectrometry

LN-229 cell lines were seeded in eight to ten 10cm cell culture plates and cultured until they reached 80% confluency. Cells were washed with 1x PBS and lyzed in 500μl 1%NP40 lysis buffer (25mM Tris pH 7.5, 150mM NaCl, 1% NP40, 0.5mM, phenylmethylsulfonylfluoride (PMSF), protease inhibitor cocktail (Roche)). Lysates were centrifuged at 13000rpm for 10minutes. Protein concentration was measured using Bradford assay (Bio-Rad Laboratories). For the first IP MS experiment 3 mg of protein was used and for the second 5 mg. The concentration of the USP15 antibody (NB110-40690, Novus Biologicals, UK) and of the normal Rabbit IgG control (12-370, Millipore, Germany) was 5μg/mg of lysate. The mixture of antibody/lysate was left rotating gently at 4°C overnight. After 12 hours 100-120μl of 50% A agarose beads (Thermoscientific, USA) were added to the samples and were incubated while rotating at 4°C for four further hours. (Before use, the beads were washed with 1xPBS and centrifuged at 1000rpm for 1 min).

The mixture (beads-antibody-protein sample) was washed 5 times with 0.1%Tween-20/PBS. (Washing: 500μl 0,1% Tween-20 PBS, mix, centrifuge for 2min at 1000rpm/4°C). The 1/10 of pulldown product: 30μl of 2X SDS-loading buffers was added, boiled for 5 min at 95°C, 5 μl of the sample were loaded onto a reducing SDS-PAGE using standard methods, and the rest was loaded on another 10% gel in parallel for silver staining (Silver Staining Kit, SilverXpress, Invitrogen; according to the manifacturer's instructions).

### Western blot analysis

For Western blot analysis protein samples were loaded onto a reducing SDS-PAGE (acrylamide, Applichem, German;, 0.33M Tris pH 8.8, 0.1M SDS, 0.1 ammonium persulfate, Biorad, USA;, TEMED, Biorad). The percentage of acrylamide was adjusted to the molecular size of the protein of interest: 8-10% for USP15, KIF15, RMDN3, and 6% for HECTD1. The samples were run for around 1h30 at 120V. Afterwards protein samples were transferred onto a nitrocellulose membrane (Amersham™Hybond™-ECL, USA). Transfer times were adjusted to the protein size: 1h30 at 300mA at 4°C for USP15, KIF15, RMDN3, and 4hours at 300mA at 4°C for HECTD1. Protein markers used for the determination of the molecular weight: peqGOLD protein marker IV, V (Peqlab, Germany) and HiMark Pre-stained HMW Protein standard (Invitrogen). After transfer, membranes were blocked in 5% milk (Rapilait) diluted in TBS-T for 1 hour in gentle rotation. Then membranes were incubated overnight at 4°C with the primary antibody (HECTD1, dilution 1:3000, NBP1-49926; KIF15, 1:3000, NBP1-49926; OSBPL3, 1:2000, NBP1-55151; RMDN3 (FAM82A2) 1:5000, NBP1-47294 - all Novus Biologicals; anti-HA antibody, 1:5000, ThermoScientific; anti-v5 tag antibody, 1:2000, ab15828, abcam; USP15 monoclonal, 1:2000, M01, clone1C10, Abnova; α-tubulin, 1:3000, T-6074, sigma). After 12hours, membranes were washed with 1xTBS-T three times and then re-incubated with the secondary antibody (ImmunoPure® Antibody, mouse, or rabbit IgG, Thermo scientific) at room temperature for 1hour, rewashed three times with 1xTBS-T. The blots were developed with Pierce ECL Western Blotting Substrate (Thermo scientific) and the signal was detected with Image Reader LAS-4000.

### Statistical analysis

The Student t test was used to compare continuous variables between two groups. P values less than 0.05 were considered statistically significant. Analyses were performed using (Prism 5, GraphPad, La Jolla, CA, USA). Results are marked with 1 asterisk (*) if p< 0.05 and 2 (^**^) if p< 0.01 and with 3(^***^) if p<0.001. All statistical tests were two-sided. Data are presented as mean values with standard deviation.

## SUPPLEMENTARY MATERIALS FIGURES AND TABLES


