# Supplementary material for: Ubiquitin Specific Peptidase 15 (USP15) suppresses glioblastoma cell growth via stabilization of HECTD1 E3 ligase attenuating WNT pathway activity
Source: Oncotarget. 2017 Nov 30;8(66):110490–502. doi: 10.18632/oncotarget.22798 (PMC5746398; doi:10.18632/oncotarget.22798)
Supplement: Supplementary file 1 [file oncotarget-08-110490-s001.pdf]

Ubiquitin Specific Peptidase 15 (USP15) suppresses glioblastoma cell growth via stabilization of HECTD1 E3 ligase attenuating WNT pathway activity

SUPPLEMENTARY MATERIALS

Cell cycle analysis

$1.5^{*}10^{6}$ cells were transfected with 50 nM of siRNAs and left to recover. After 24 hours of culture with 5% FCS in DMEM media, cells were subjected to serum starvation (0.1% FCS) for 48 hours to synchronize cells in G1 phase. After serum starvation cells were stimulated by adding 5% FCS to the media for 24 hours to enter the cell cycle. Cells were washed twice with 1x PBS and harvested by trypsinization (Gemco, UK). Cells were fixed with 70% ethanol and left at 4°C overnight. The next day, they were washed with 1xPBS and then subjected to RNAase A treatment and stained with Propidium Iodide (PI) (Sigma) for 30 min at room temperature and analyzed by fluorescence-activated cell sorting (FACS). Cell size was evaluated by forward scatter (FS) and side scatter (SS) analysis, and doublets were excluded using pulse area/pulse width. Cells were analysed with a BD LSR II system. 5×10^{4} events were counted for all samples. FACS cell cycle data were analyzed by ModFit LT™ software.

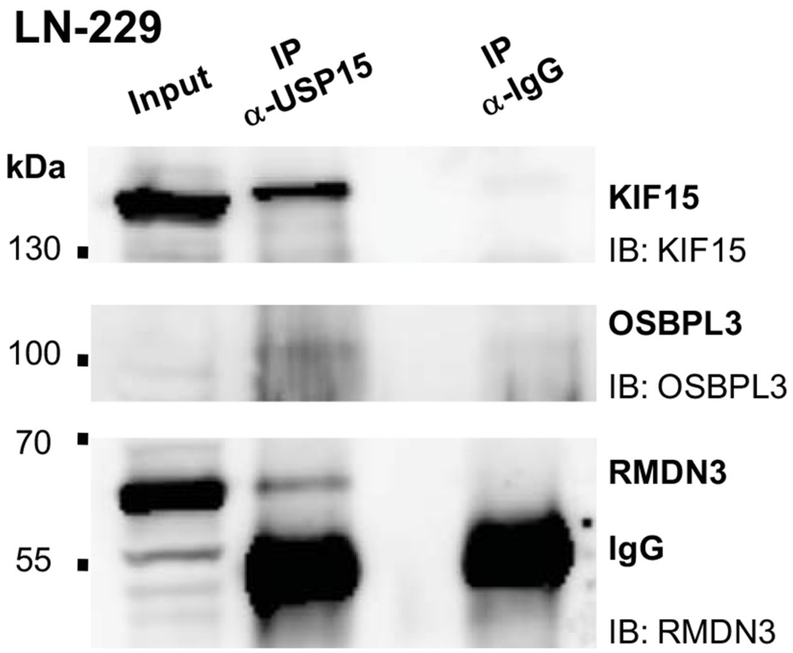

Supplementary Figure 1: USP15 interactions in the GBM cell line LN-229. Immunoprecipitation using an antibody against the endogenous USP15 in LN-229 protein extract, analyzed with Western blot using antibodies against KIF15 (dilution 1:3000, NBP1-49926, Novus Biologicals), OSBPL3 (dilution 1:2000, NBP1-55151, Novus Biologicals), and RMDN3 (FAM82A2, 1:5000, NBP1-47294, Novus Biologicals).

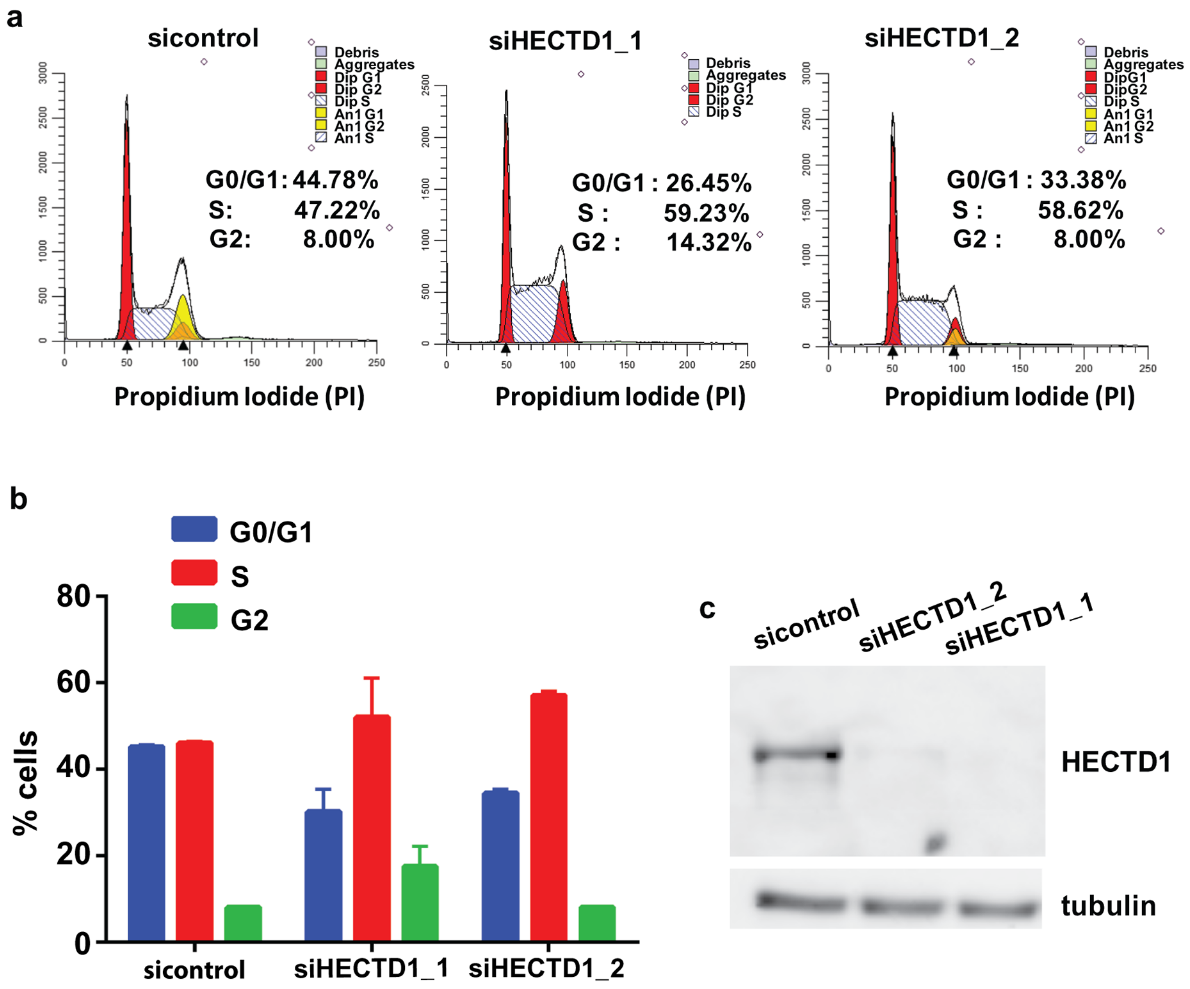

Supplementary Figure 2: HECTD1 knockdown in LN-229 cells. LN-229 cells were transfected with 50 nM of siHECTD1_1, siHECTD1_2, or sicontrol (for sequences see Supplementary Table 2) and were left to recover for 24 hours. After synchronization of the cells in G0/G1 by serum starvation, cells were stimulated with 5% serum for 24 hours. Cells were harvested, stained with propidium iodide (PI) and analyzed by FACS. Depletion of endogenous HECTD1 lead to a faster transition of LN-229 cells from G0/G1 to S phase promoting the cell cycle **(a)**. The quantification the experiments is visualized in a histogram represented as mean +/- SD **(b)**. HECTD1 knockdown was confirmed by Western blot 96 hours after transfection **(c)**. (See Supplementary Method for details)

Supplementary Table 1: USP15 Interactors of the *STRIPAK complex in LN-229

Identifier	UniProt description	Number of Spectra			
		1st IP		2nd IP	
		α-USP15	IgG control	α-USP15	IgG control
**USP15**	Ubiquitin carboxyl-terminal hydrolase 15	**232**	**2**	**392**	**0**
**HECTD1**	E3 ubiquitin-protein ligase HECTD1	**25**	**0**	**68**	**0**
**STRN4**	Striatin-4	**2**	**1**	**4**	**0**
**STRN3**	Striatin-3	**9**	**1**	**15**	**0**
**FAM40A**	Protein FAM40A	**-**	**-**	**4**	**0**
**STRN**	Striatin	**-**	**-**	**4**	**0**
**CTTNBP2**	Cortactin-binding protein 2	**-**	**-**	**5**	**0**
**CTTNBP2NL**	CTTNBP2 N-terminal-like protein	**-**	**-**	**5**	**0**

* striatin-interacting phosphatase and kinase.

Supplementary Table 2: Sequences of siRNAs and primers

siRNAs		
**Gene/Name**	**Sense strand sequence (core seq.) 5'-3'**	
**USP15_1**	GAUGAUACCAGGCAUAUAA	
**USP15_2**	GGUAUUGUCCGAAUUGUAA	
**USP15_3**	Mission esiRNA, EHU0 90661-20 UG, Sigma	
**HECTD1_1**	AGAUAAAGGUGGUGAUAUA	
**HECTD1_2**	GAGAACACUUGGAGAGAUU	
**Neg. control**	AGGUAGUGUAAUCGCCUUGtt	
**PrImers**		
**Gene/Name**	**Forward 5'-3'**	**Reverse 5'-3'**
***USP15* residues 1–1627**	AAAAGTCGACAT GGCGGAAGGCGGAGCG	GCGCGGTAACTAT CATCTTATCTGCAGGTATTCC
***USP15* residues 1628-3000**	GCGCGCAATACCTGC AGATAAGATGATAGTTACTGA	GCGCTTCTAGATTA GTTAGTGTGCATACAGTTTTC
***USP15*C/S**	ATAGAGGCCTCTGTGGCCTAAGTAAC TTGGGAAATACGAGTTTC	GCTGAGTTCATG AAACTGTATTTCCCAAGTTAC
**USP15**	CAACCACTATGGAGGGATGG	TTGGTCTTCAG ATGCAGTGG
**USP15_2**	ACTGAGGATACTTG CAAAGGTCAACTC	CATCAAATCTTATATG CCTGGTATCATC
**HECTD1**	CATCAAATCTTATA TGCCTGGTATCATC	GCGGCTTCTTGG GAACTCTA
**AXIN2**	GAGACCCAGCAGCACCTTTC	CAATGGCAAAC AGAATGTACAGATT
